# Clinical Outcomes of Subacromial Injections With Hyaluronic Acid for the Treatment of Tendinopathies and Partial Supraspinatus Tears

**DOI:** 10.7759/cureus.82376

**Published:** 2025-04-16

**Authors:** Mauro E Gracitelli, Leonardo Zanesco, Caio Checchia, Isabela Assad, Lucas Nunes Lino da Silva, Jorge Assunção, Thais Vasques, Eduardo A Malavolta

**Affiliations:** 1 Orthopedics, Hospital das Clínicas da Faculdade de Medicina da Universidade de São Paulo, São Paulo, BRA; 2 Orthopedics, Hospital do Coração (HCor), São Paulo, BRA; 3 Orthopedics, Hospital Sírio-Libanês, São Paulo, BRA; 4 Physical Therapy, Faculdade de Medicina da Universidade de São Paulo, São Paulo, BRA; 5 Orthopedics, Hospital das Clínicas HCFMUSP, Faculdade de Medicina, Universidade de São Paulo, São Paulo, BRA

**Keywords:** chronic tendinopathy, injection hyaluronic acid, rotator cuff pathology, rotator cuff tears, shoulder joint pain

## Abstract

Background

Rotator cuff syndrome, including tendinopathy and partial-thickness tears, is a common cause of shoulder pain. While conservative management remains the first-line treatment, subacromial hyaluronic acid (HA) injections have been proposed as an alternative for patients with persistent symptoms.

Methods

This retrospective case series evaluated the clinical outcomes of subacromial HA injections in adults diagnosed with rotator cuff syndrome, presenting with tendinopathy or partial-thickness tears on MRI. Patients received two HA injections, two weeks apart. The primary outcome was the improvement in the American Shoulder and Elbow Surgeons (ASES) score. Secondary outcomes included the Single Assessment Numeric Evaluation (SANE) score and pain reduction using the visual analog scale (VAS). Assessments were conducted at baseline and at one, three, six, and 12 months post-injection.

Results

A total of 41 patients (68 shoulders) were included, with a mean age of 50.1 ± 14.5 years; 53.6% were female. The ASES score improved from 45.5 ± 17.2 at baseline to 63.4 ± 25.1 at 12 months (p < 0.001). The SANE score increased from 61% ± 17 to 76% ± 19 (p = 0.013), while VAS decreased from 6.3 ± 1.9 to 4.3 ± 3.0 (p < 0.001). There was no difference in the ASES, SANE, and VAS scores between the tendinopathy and partial tear groups at any moment.

Conclusion

Subacromial HA injections resulted in clinically and statistically significant improvement in ASES, SANE, and VAS scores at 12 months. The treatment provided consistent symptom relief and functional improvement over time, with no reports of complications.

## Introduction

Rotator cuff syndrome, which manifests as tendinopathy or tendon tears, is the leading cause of shoulder pain [1-3]. For rotator cuff injuries lacking full-thickness tears, conservative treatment modalities are predominantly employed [1].

Subacromial injections comprising anesthetics and corticosteroids (CS) are commonly used for patients with persistent symptoms despite rehabilitation and nonsteroidal anti-inflammatory drugs (NSAIDs) [4,5]. This approach yields superior short-term outcomes compared to placebo [5,6]. However, there are long-term risks associated with CS injections, such as reduced tendon cell proliferation and decreased mechanical strength [7].

Recent studies have shown promising results regarding hyaluronic acid (HA) injections for rotator cuff treatment [4,5,8]. HA's theoretical advantages for the rotator cuff encompass anti-inflammatory and anti-adhesive characteristics, along with its capacity to suppress cyclooxygenase-2 production of prostaglandin E2 by subacromial fibroblasts [9,10].

Despite four randomized studies [11-14] comparing HA with placebo or CS, no previous study has evaluated the use of phosphate-buffered HA (PB-HA). This is the only HA formulation approved by a local regulatory agency for soft tissue applications. Investigations on this type of HA for lateral epicondylitis have demonstrated superior efficacy compared to placebo [15].

This study evaluates the clinical outcomes of subacromial HA injections in adults diagnosed via MRI with rotator cuff tendinopathy or partial-thickness tears. The objective is to contribute further evidence regarding the efficacy and safety of HA injections in the management of rotator cuff-related pain and dysfunction.

## Materials and methods

Objectives

The present research aims to evaluate the clinical outcomes of subacromial HA injections in adult patients diagnosed with rotator cuff syndrome, presenting with tendinopathy or partial-thickness tears on MRI exam. The primary outcome is the improvement in the American Shoulder and Elbow Surgeons (ASES) score [16]. The secondary outcomes included the assessment of shoulder functionality and patient satisfaction through the Single Assessment Numeric Evaluation (SANE) score [17], as well as pain relief measured through the visual analog scale (VAS) [18]. Additionally, this study will monitor and document any adverse events associated with the administration of HA injections.

Study design

This study is a case series of adult patients with rotator cuff syndrome who received subacromial HA injections. Data were extracted from electronic health records at a single orthopedic center, covering the period from 2020 to 2024. The study was approved by the institutional ethics committee (CAAE: 32003320.8.0000.0068). Data pertaining to the characteristics of the lesions were acquired through magnetic resonance imaging (MRI) and categorized as tendinopathy or partial-thickness tears, which were further divided into bursal, interstitial, and articular-sided tears. Demographic and clinical data of the participants were also collected, encompassing age, gender, weight, height, limb dominance, affected side, smoking status, diabetes, hypertension, work-related issues, and history of trauma or previous surgery.

Participants

The study included patients aged 18 years and older with at least two months of shoulder pain, exhibiting clinical signs of subacromial pain, and MRI findings consistent with tendinopathy or partial-thickness tear of the supraspinatus tendon.

The exclusion criteria were passive limitation of shoulder range of motion, full-thickness rotator cuff tear (regardless of the tendon involved, tear size, or retraction), history of fracture or dislocation in the affected shoulder, glenohumeral osteoarthritis or focal cartilage lesions in the same shoulder, neurological injury in the ipsilateral limb, any previous surgery on the ipsilateral upper limb, cognitive impairment affecting comprehension of questionnaires, active or previous infection in the affected shoulder, and inability to maintain follow-up after injection.

Intervention

Patients received two subacromial HA injections, two weeks apart. The HA used was Sportvis® (Pharmascience Inc., Montreal, Canada) (12 mg in 1.2 mL of 1% sodium hyaluronate in a PB solution, biocompatible for periarticular injection into soft tissues, as per the manufacturer’s instructions).

All injections were performed in the subacromial space (between the acromion and the supraspinatus tendon) by experienced orthopedic surgeons using anatomical landmarks. The procedure was conducted under standard aseptic conditions with cutaneous antisepsis and a local anesthetic skin wheal utilizing 2% lidocaine without a vasoconstrictor. The HA injection was subsequently administered using a 30 × 7 mm needle. Following the injection, patients were instructed to utilize the affected limb within normal parameters but to refrain from strenuous activities.

After the initial injection, a regimen of comprehensive shoulder stretching exercises was prescribed as part of physical therapy. Subsequent to the second injection, a one-week interval preceded the introduction of strengthening exercises targeting the rotator cuff, deltoid, and periscapular musculature. Clearance for participation in sports or physical activities was granted two weeks post-second injection or as individually tolerated by each patient. All patients received a standardized regimen of non-steroidal anti-inflammatory drugs and non-opioid analgesics.

Outcomes

The evaluated outcomes encompassed the ASES Standardized Shoulder Assessment Form as the primary outcome, in conjunction with the SANE and the VAS as secondary outcomes. The minimally clinically important difference (MCID) for the ASES score was established at 15 points, with an anticipated standard deviation of 20 [19]. Complications, including allergic reactions and infections, were documented throughout the follow-up period to evaluate the safety profile of the intervention.

Patients underwent evaluation at baseline and subsequent follow-up assessments at one, three, six, and 12 months post-initial injection, with the 12-month assessment serving as the primary endpoint for the study. Data collection encompassed clinical examinations and the completion of ASES, SANE, and VAS questionnaires at each follow-up visit.

Statistical analysis

Continuous variables were assessed for normality utilizing the Shapiro-Wilk test, which indicated the necessity for non-parametric methods due to the non-normal distribution of the data. Continuous data were presented as mean and standard deviation and correlated using Pearson's coefficient. Categorical variables were expressed as absolute and percentage values and analyzed using ANOVA or Kruskal-Wallis tests. Sequential time-point comparisons were conducted using the Friedman test, followed by Bonferroni post-hoc analysis to determine the level of significance adjusted multivariate analysis. The Mann-Whitney U test was employed to assess differences between subgroups with partial-thickness tears and tendinopathy. 

A significance level of p < 0.05 was considered for all statistical tests. Statistical analyses were conducted using Google Colab (Google, Inc., Mountain View, CA) (using Python version 3.10, Guido van Rossum, Centrum Wiskunde and Informatica (CWI), Amsterdam, the Netherlands), employing libraries such as pandas (version 1.5.3, Wes McKinney, AQR Capital, Greenwich, CT) for data manipulation and SciPy (version 1.10.1, Travis Oliphant, Rochester, MN) for statistical computations.

## Results

A total of 68 shoulders from 41 patients were evaluated, comprising 19 men (47%) and 22 women (53%), with a mean age of 50.1 ± 14.5 years. Among these, 55.9% presented with partial-thickness tears, while 44.1% exhibited tendinopathy. The majority of procedures were performed on the dominant side (75.6%). Patients' demographics are summarized and presented in Table 1.

**Table 1:** Cohort demographic characteristics

	N	%
Female gender	22	53.6
Dominant side affected	10	24.4
Diabetes mellitus	8	19.5
Hypertension	7	17.1
Smoking	7	17.1
Labor litigation	5	12.2

Clinical outcomes

The ASES scores demonstrated a statistically significant improvement, from a mean (SD) of 45.5 (±17.2) pre-treatment to 63.4 (± 25.1) at 12 months of follow-up (p < 0.001). The SANE scores improved from 61% initially to 76% at 12 months (p = 0.013), and the VAS scores exhibited a reduction from 6.3 ± 1.9 to 4.3 ± 3.0 (p < 0.001), both with statistical significance. 

**Figure 1:**
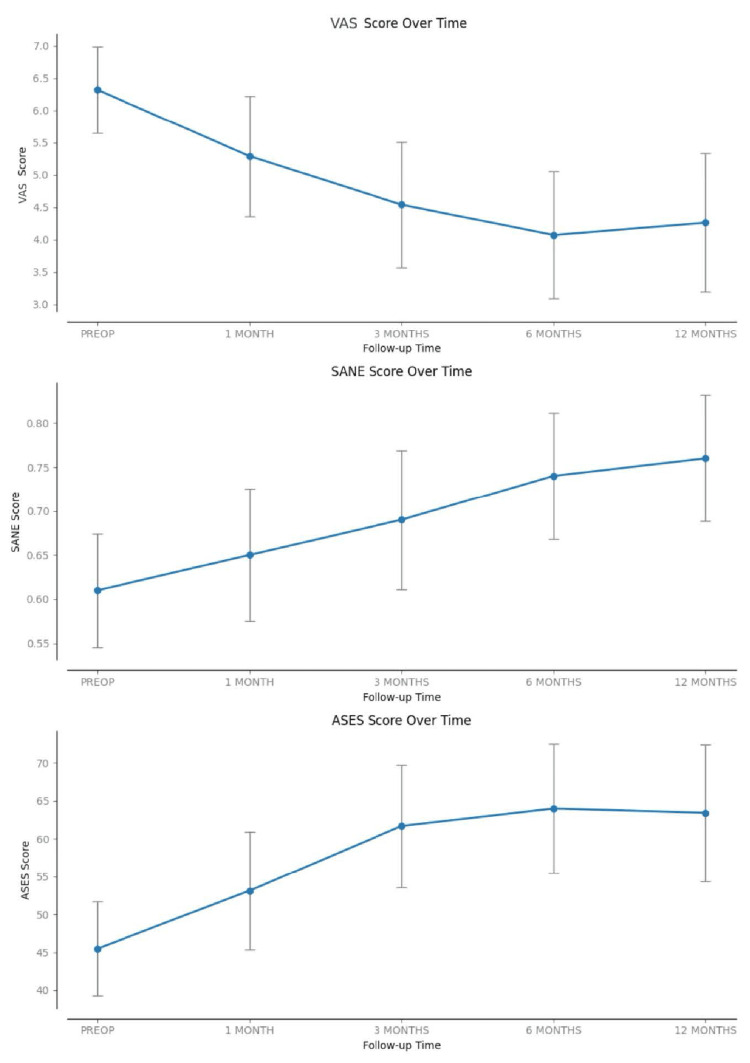
Evolution of scores over time Error bars represent the 95% confidence intervals. ASES: American Shoulder and Elbow Surgeons Score; SANE: Single Numeric Assessment Evaluation; VAS: visual analog scale

Temporal analysis (Figure 1) shows consistent improvement over time. At the three-month mark, the ASES score was 61.7 (± 22.5, p < 0.001), already achieving MCID. VAS scores also revealed significant pain improvement at this time point, 4.5 (± 2.7, p < 0.001). While overall improvement was observed over time, not all consecutive time points demonstrated statistically significant differences, indicating a certain plateau, especially toward the second half of the study.

No patient had complications related to the procedure, such as infection or skin complications.

Subgroup analyses

Both at baseline and after 12 months, a comparison between the partial-thickness tear and tendinopathy subgroups did not reveal any statistically significant differences. The final VAS score was 4.1 for the tendinopathy group and 4.0 for the partial-thickness tear group (p = 0.960). Similarly, the ASES and SANE scores demonstrated no significant differences between the two groups (p = 0.936 and p = 0.950, respectively) (Table 2).

**Table 2:** Tendinopathy and partial tear subgroup results comparison p-value obtained from the Mann-Whitney U test.

	ASES (mean ± SD)		SANE (% ± SD)		VAS (mean ± SD)	
	Baseline	12 months	Baseline	12 months	Baseline	12 months
Tendinopathy	45.13 ± 20.1	64.38 ± 23.4	61.8% ± 19.5	74.3% ± 21.9	6.1 ± 1.7	4.0 ± 3.1
Partial tear	46.75 ± 16.0	63.67 ± 24.4	60.3% ± 16.3	73.4% ± 19.4	6.5 ± 1.9	4.1 ± 2.4
p	0.617	0.935	0.920	0.950	0.313	0.960

## Discussion

The present study investigated the effects of subacromial HA injections in patients with rotator cuff syndrome, focusing on pain reduction and functional improvement over a 12-month follow-up period. The results demonstrated a statistically significant improvement in the ASES, SANE, and VAS scores. Subgroup analyses revealed no statistically significant differences between patients with tendinopathy and those with partial-thickness tears at 12 months, suggesting a similar clinical response to HA injections across these diagnostic categories.

The present findings are consistent with the results from previous randomized clinical trials that have investigated the effects of HA injections on rotator cuff-related pathologies. For example, Chou et al. [11] conducted a double-blind, placebo-controlled trial involving 51 patients with rotator cuff lesions without complete tears. This study demonstrated a significant reduction in the VAS score from 6.36 ± 1.35 at baseline to 3.04 ± 2.03 six weeks after subacromial HA injections, compared to an improvement from 6.46 ± 1.27 to 5.12 ± 2.42 in the placebo group (p = 0.018). At a 33.1-month mean follow-up, patients who had undergone HA injections exhibited a mean VAS score of 1.53 ± 1.62. A randomized controlled trial (RCT) by Rezasoltani et al. [20] also compared low-molecular-weight HA with physiotherapy in 51 patients with supraspinatus tendinopathy, revealing significantly greater reductions in VAS scores at rest (3.48 ± 2.58 vs. 1.17 ± 1.47, p < 0.001) and better overall shoulder motion at three months in the HA group. This aligns with our finding that HA injections effectively reduce pain over time. While our results indicate a generally higher final VAS score, this observation holds true only when compared to a longer follow-up period or to a substantially lower baseline pain score. 

Cai et al. [21] evaluated the use of HA, platelet-rich plasma (PRP), a combination of both, and no injections in 184 patients with partial-thickness rotator cuff tears. Although their study reported higher scores for the groups that received PRP, the 12-month ASES score was significantly higher (p < 0.05) for the HA group (60.93 ± 4.65) compared to the non-injection group (47.89 ± 2.56). These findings are consistent with our research group's results, in which our patients' ASES score improved from 45.5 (± 17.2) to 63.4 (± 25.1).

In a prospective study by Cipolletta et al. [22], patients receiving ultrasound-guided HA injections demonstrated a reduction in the VAS pain scores from a mean (SD) of 6.2 ± 1.5 at baseline to 2.2 ± 2.0 at 3 months post-treatment (p < 0.01). Similarly, a randomized controlled trial by Mohebbi et al. [23] observed significant pain reduction after three months; however, their findings are less directly comparable as they categorized pain separately at rest, at night, and during activity. Nevertheless, both studies corroborate our findings, suggesting that HA injections may be an effective strategy for achieving expedited pain relief in patients with rotator cuff tendinopathy and partial tears.

It is also worth mentioning that patients included in this study were predominantly refractory to conservative treatment, having previously undergone physical therapy without significant improvement. Despite persistent symptoms, they did not meet surgical criteria due to the absence of full-thickness rotator cuff tears. This represents a more complex patient profile, wherein additional factors such as individual pain perception [24] and psychosocial characteristics [25,26] that have been demonstrated to negatively influence the final outcome undoubtedly contribute to the overall clinical picture. Additionally, while surgical repair typically results in ASES scores exceeding 80 points at two years [27], our findings showed lower post-treatment scores, aligning more closely with preoperative values seen in surgical candidates.

The present study exhibits some limitations. Firstly, the retrospective design inherently introduces the potential for selection bias and incomplete data collection. Although patients had well-defined inclusion criteria, the absence of a control group limits the capacity to draw definitive causal conclusions regarding the efficacy of HA injections. Secondly, the relatively small sample size (68 shoulders) diminishes the statistical power for subgroup analyses, particularly when evaluating differences between tendinopathy and partial-thickness tear groups; however, the sample size was similar to that of previously published studies. In addition, given the increasing availability of point-of-care ultrasound (POCUS), future studies should consider incorporating image-guided injections to reduce variability in delivery.

Despite these limitations, the findings of this study have shown the potential benefits of HA injections in a challenging subset of patients with rotator cuff syndrome. Over time, there was a consistent improvement in pain and function scores across both the tendinopathy and partial-thickness tear subgroups when conventional rehabilitation modalities had failed. Further prospective, randomized studies are warranted to corroborate these findings and investigate the long-term outcomes associated with HA injections.

## Conclusions

Subacromial HA injections resulted in clinically and statistically significant improvement in ASES, SANE, and VAS scores at 12 months. The treatment provided consistent symptom relief and functional improvement over time, with no reports of complications.
